# Essentials of Anatomy and Manual of Practical Dissection

**Published:** 1891-12

**Authors:** 


ESSENTIALS OF ANATOMY AND MANUAL OF PRACTICAL DISSECTION, TOGETHER WITH THE ANATOMY OF THE VISCERA. Prepared especially for Students of Medicine. By CHARLES B. NANCREDE, M. D., Professor of Surgery and of Clinical Surgery in the University of Michigan, Ann Arbor; Corresponding Member of the Royal Academy of Medicine, Rome, Italy; late Senior Surgeon to Episcopal Hospital; late Surgeon to Jefferson Medical College Hospital; formerly Lecturer on Osteology, etc., in Medical Department, University of Pennsylvania; late Professor of General Orthopedic Surgery in Philadelphia Polyclinic, etc. Fourth edition, revised and enlarged by an Appendix containing Hints on Dissection, by J. CHALMERS DA-COSTA, M. D., based upon the last edition of Gray's Anatomy. Thirty handsome full-page lithographic plates in colors, and 188 fine wood-cuts. Philadelphia: W. B. Saunders, 913 Walnut street. 1891.

The usefulness of this book to the medical student pursuing his anatomical course, can scarcely be overstated. The practical hints on dissection are so clear and simple that it seems to us that no one of ordinary intelligence can fail to follow them with ease. Thirty beautifully executed colored lithographs illustrate the most important parts in medical or surgical points of view. Taken all in all, nothing appears to be wanting to make it an excellent companion to the scalpel at the dissecting table. The larger part of the book is devoted to what its author calls the Essentials of Anatomy, an arrangement in the form of questions and answers, and it is safe to say that the student who can answer all these questions correctly, may fearlessly face the examiner in anatomy when the crucial trial comes. Excellent wood-cuts illustrate the text.

